# Proton pump inhibitor treatment is associated with acute-on-chronic liver failure in patients with advanced cirrhosis

**DOI:** 10.1097/HC9.0000000000000178

**Published:** 2023-06-22

**Authors:** Lukas Sturm, Chiara Gahm, Michael Schultheiss, Marlene Reincke, Jan Patrick Huber, Tobias Boettler, Robert Thimme, Dominik Bettinger

**Affiliations:** 1 Department of Medicine II, Medical Center University of Freiburg, University of Freiburg, Freiburg, Germany; 2 Berta-Ottenstein-Program, University of Freiburg, Freiburg, Germany

## Abstract

Acute-on-chronic liver failure (ACLF) is a fatal complication of cirrhosis. Hence, identification of risk factors for ACLF is crucial. Previous studies have linked proton pump inhibitor (PPI) treatment to complications of cirrhosis, however, a possible effect of PPI treatment on the risk of ACLF has not been investigated yet. Therefore, the present study aimed to characterize the impact of PPI treatment on ACLF development.

**Methods:**

A total of 642 patients hospitalized due to complications of cirrhosis were retrospectively identified, and PPI treatment during an observation period of 3 years following the hospitalization was reviewed. Subsequently, 74 patients with newly initiated PPI treatment at the time of hospitalization (PPI group) were 1:1 propensity score matched to 74 patients who received no PPI treatment (no-PPI group). Primary end point was the development of ACLF during the observation period, and secondary endpoints were mortality and upper gastrointestinal bleeding.

**Results:**

PPI and no-PPI groups had comparably severe chronic liver disease at baseline. Nevertheless, the cumulative incidence of ACLF in the presence of death as competing risk was markedly higher in the PPI group compared with the no-PPI group. ACLF-related deaths contributed significantly to a higher 3-year mortality in the PPI group. Uni and multivariable competing risk regression models confirmed that PPI treatment was an independent predictor of ACLF in the study collective (subdistribution HR: 1.892, 95% CI: 1.092–3.281, *p* = 0.023). The impact of PPI treatment on ACLF development was particularly strong in patients with a model for end-stage liver disease score >12. Upper gastrointestinal bleeding was slightly less frequent in the PPI group.

**Conclusions:**

The present results indicate that PPI treatment could be a risk factor for ACLF in patients with advanced cirrhosis.

## Graphical abstract

**Figure:**
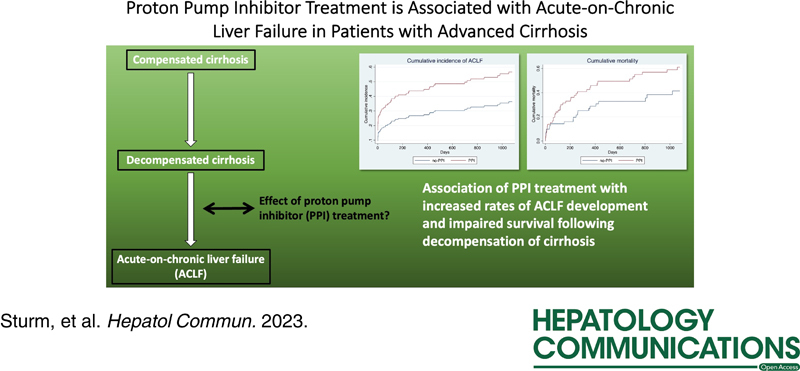

## INTRODUCTION

In recent years, acute-on-chronic liver failure (ACLF) has emerged as a distinct syndrome in cirrhosis characterized by multi-organ failure. ACLF is a severe complication, as it is associated with high morbidity and mortality.^1^ Hence, the identification of risk factors for the development of ACLF is of major importance. A number of previous studies have demonstrated an association of proton pump inhibitor (PPI) intake with complications of cirrhosis, such as HE and spontaneous bacterial peritonitis (SBP).^2–6^ The pathomechanisms underlying the association of PPI treatment with complications of cirrhosis are subject to ongoing research. However, a number of studies suggest that PPI treatment, especially in the long term, induces intestinal dysbiosis, which may aggravate bacterial translocation from the gut and enhance the chronic inflammatory state characteristic of advanced cirrhosis.^6–9^ Of note, an inflammatory status is a typical finding in patients developing ACLF.^1,10^ Against this background, we hypothesized that PPI treatment could be a predisposing factor for the development of ACLF. Therefore, the aim of the present study was to investigate if PPI treatment is a risk factor for ACLF in a propensity score matched collective of patients with cirrhosis.

## METHODS

### Patient selection

**FIGURE 1:**
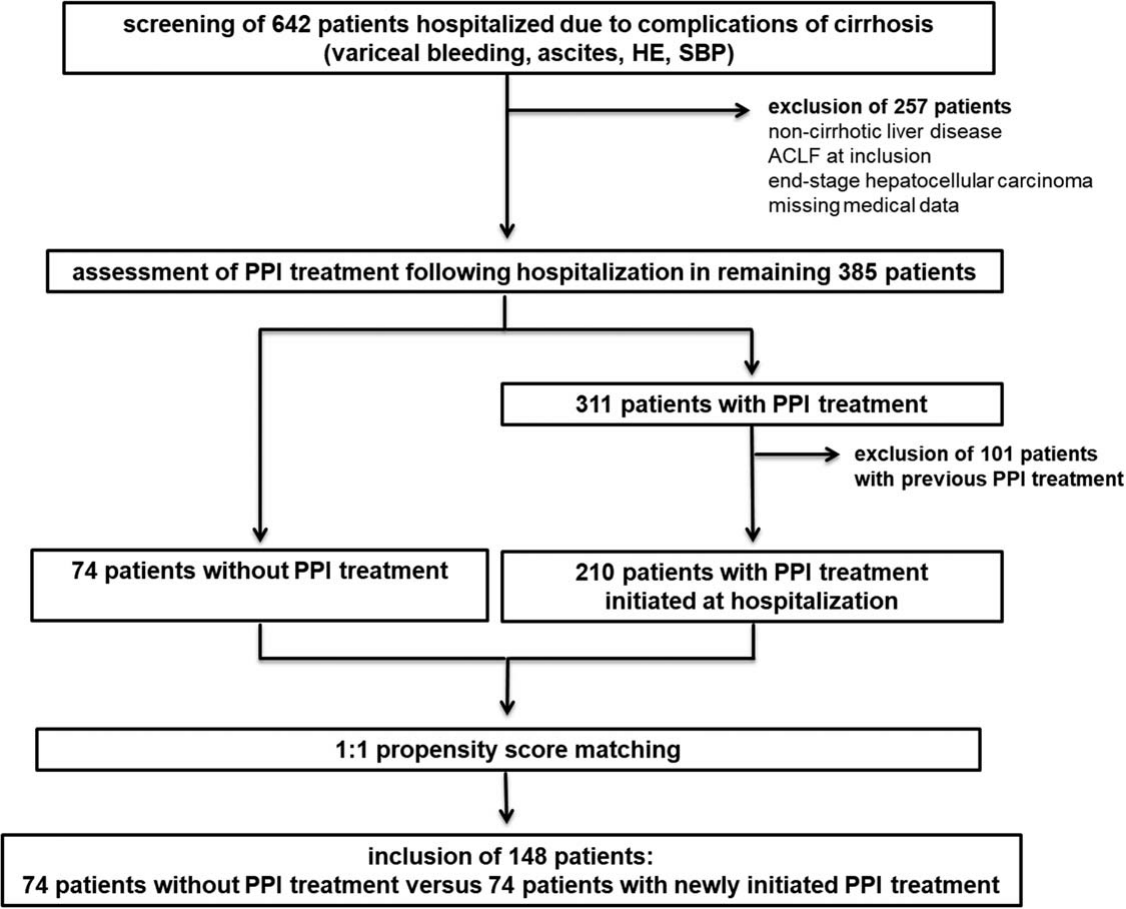
Patient selection. A total of 642 patients with cirrhosis hospitalized due to complication of cirrhosis (variceal bleeding, ascites, HE, and SBP) were retrospectively identified. However, 257 patients with non-cirrhotic portal hypertension, ACLF, end-stage HCC, or missing medical data were excluded. The remaining 385 patients were stratified according to whether they received PPI treatment during an observation period of 3 years following the hospitalization. Only patients in whom PPI treatment was newly initiated during the index hospitalization were considered for study inclusion. This left 74 patients without PPI treatment and 210 patients with PPI treatment who entered 1:1 propensity score matching. Finally, 148 patients were included in the analyses: 74 patients without PPI treatment and 74 matched patients with PPI treatment. Abbreviations: ACLF, acute-on-chronic liver failure; PPI, proton pump inhibitor; SBP, spontaneous bacterial peritonitis.

Patient selection is summarized in Figure 1. A total of 642 patients hospitalized due to complications of cirrhosis at the University Medical Center Freiburg, Germany, between January 2005 and January 2019 were retrospectively identified. The following complications were recorded: variceal bleeding, ascites, HE, and SBP. Overall, 257 patients with non-cirrhotic chronic liver disease, ACLF according to the criteria of the European Association for the Study of the Liver (EASL),^1^ end-stage HCC (Barcelona clinic liver cancer stage *D*), or missing medical data were excluded. In the remaining 385 patients, PPI medication during an observation time of 3 years following the initial hospitalization was reviewed in the medical records. To avoid bias related to PPI-exposed time, only patients in whom PPI treatment was initiated at the time of hospital admission were considered for study inclusion. Hence, 101 patients who received PPI treatment before the index hospital stay were excluded. This left 210 patients with PPI treatment and 74 patients without PPI treatment that entered propensity score matching. In patients with PPI treatment, indication for PPI therapy, substance, and daily dose were noted, whereby pantoprazole served as reference (esomeprazole and omeprazole dose equaled double the pantoprazole dose). Further, new initiation, discontinuation, or dose changes of PPI treatment during the observation period were assessed. Changes in PPI therapy were recorded when first registered in the medical records.

### Propensity score matching

Subsequently, the 74 patients without PPI treatment (no-PPI group) were 1:1 propensity score matched to 74 patients with PPI treatment (PPI group), which resulted in a total of 148 patients enrolled in the analyses. Matching criteria were model for end-stage liver disease (MELD) score as a measure of laboratory liver function and clinical presentation of decompensated cirrhosis at study inclusion (variceal bleeding, ascites, HE, and SBP). Maximum caliper width for matching was ±0.02. The main results of the study were validated internally by performing 1:2 propensity score matching with less strict matching criteria, which resulted in a larger cohort of 222 patients (n = 74 without PPI treatment vs n = 148 with PPI treatment).

### Data collection

The study was an observational analysis. The included patients’ demographic, clinical, laboratory, interventional, radiologic, and survival data were assessed in the medical records. Baseline parameters were recorded at the date of hospital admission. Primary end point was the development of ACLF within 3 years after the index hospitalization. In this context, the following defined precipitants of ACLF were identified: hemorrhagic shock, severe alcohol-associated hepatitis, toxic encephalopathy, and bacterial infections.^11^ Secondary end points were 3-year mortality and the occurrence of upper gastrointestinal bleeding.

### Ethics statement

The study was approved by the local ethics committee (no. EK 21-1074) and is in accordance with the Declaration of Helsinki and Declaration of Istanbul. Due to the retrospective nature of the study, informed patient consent was waved. The study was conducted in accordance with the STROBE guidelines.^12^

### Statistical analyses

Categorical variables were expressed as frequencies and percentages and continuous variables as median with interquartile range. Differences between PPI group and no-PPI group were determined by chi-square tests in case of categorical variables and by Wilcoxon rank-sum tests in case of continuous variables, as there was no Gaussian distribution of the data. *P* values < 0.05 were considered significant. The development of ACLF in the presence of death as competing risk was explored by calculating the cumulative incidence function. Predictors of ACLF were analyzed by fitting uni- and multivariable Fine & Gray competing risk regression models.^13^ In this context, PPI treatment and daily dose were modeled as time-varying covariates to consider changes in PPI therapy during the observation period. Variables that were identified as predictors of ACLF in the PREDICT study^10^ or with a *p* value < 0.10 in univariable regression were entered into multivariable regression analyses. Statistical analyses were performed with SPSS (Version 28.0, IBM), GraphPad Prism (Version 9.3, GraphPad Software), and STATA (Version 17.0, Stata Corp Lp.).

## RESULTS

### Patient baseline characteristics

Table 1 summarizes patient characteristics at baseline. Propensity score matching yielded 2 well-matched patient groups. There were no significant group differences with respect to age, sex distribution, and etiology of liver disease, with alcohol-associated liver disease being the leading cause of cirrhosis. PPI group and no-PPI group were in comparably advanced stages of cirrhosis as assessed by the MELD score, chronic liver failure-consortium acute decompensation score (CLIF-C AD), and Child-Pugh-Turcotte stage. PPI and no-PPI groups were also comparable with regard to the clinical presentation of cirrhosis at study inclusion, as there were no significant group differences with respect to the number of patients with variceal bleeding, ascites, HE, and SBP. A small proportion of patients in both groups had HCC. Further, the severity of comorbidities was comparable in PPI and no-PPI groups, determined by the updated Charlson Comorbidity Index. A vast majority of patients in the PPI group were prescribed pantoprazole with a daily dose of 80 mg or 40 mg at study inclusion. PPI treatment was initiated during the observation period in 2 patients in the no-PPI group (after 132 d and 899 d, respectively). Discontinuation of PPI treatment was recorded in none of the patients in the PPI group. However, daily PPI dose was changed in 11 patients (reduction in 10 patients and increase in one patient, after 203 (48–695) days). Of note, in a significant proportion of patients in the PPI group, no clear indication for PPI therapy could be determined. Baseline characteristics of the 1:2 propensity score matched internal validation collective are summarized in (Supplemental Table S1, http://links.lww.com/HC9/A321).

**TABLE 1:** Baseline characteristics of the 1:1 propensity score matched cohort

	Total of included patients (n = 148)	No-PPI group (n = 74)	PPI group (n = 74)	*p*; no-PPI vs PPI group	Unmatched PPI-treated patients (n = 136)
Sex				0.715	—
Female	42 (28.4)	20 (27.0)	22 (29.7)	—	39 (28.7)
Male	106 (71.6)	54 (73.0)	52 (70.3)	—	97 (71.3)
Age (y)	62 (52–71)	60 (51–69)	64 (52–71)	0.273	58 (49– 67)
Etiology				0.652	—
Alcohol-associated	71 (48.0)	32 (43.2)	39 (52.7)	—	74 (54.4)
HBV	8 (5.4)	5 (6.8)	3 (4.1)	—	3 (2.2)
HCV	18 (12.2)	10 (13.5)	8 (10.8)	—	19 (14.0)
NASH	7 (4.7)	2 (2.7)	5 (6.8)	—	7 (5.1)
Autoimmune	14 (9.5)	8 (10.8)	6 (8.1)	—	9 (6.6)
Other/cryptogenic	30 (20.3)	17 (23.0)	13 (17.6)	—	24 (17.7)
HCC	25 (16.9)	15 (20.3)	10 (13.5)	0.273	17 (12.5)
BCLC stage				0.101	—
A	5 (20.0)	4 (26.7)	1 (10.0)	—	3 (17.6)
B	11 (44.0)	4 (26.7)	7 (70.0)	—	12 (70.6)
C	9 (36.0)	7 (46.7)	2 (20.0)	—	2 (11.8)
Variceal bleeding	40 (27.0)	20 (27.0)	20 (27.0)	0.999	122 (89.7)^**a**^
Ascites	107 (72.3)	51 (68.9)	56 (75.7)	0.358	98 (72.1)
HE	32 (21.6)	17 (23.0)	15 (20.3)	0.690	32 (23.5)
SBP	14 (9.5)	6 (8.1)	8 (10.8)	0.574	8 (5.9)
MELD	13 (10–18)	13 (10–17)	14 (10–19)	0.192	14 (10–20)^**a**^
CLIF-C AD	49 (43–58)	47 (42–57)	50 (43–58)	0.201	52 (45–61)^**a**^
Child-Pugh-Turcotte stage				0.384	—
A	34 (23.0)	20 (27.0)	14 (18.9)	—	17 (12.5)^**a**^
B	72 (48.6)	36 (48.6)	36 (48.6)	—	61 (44.9)
C	42 (28.4)	18 (24.3)	24 (32.4)	—	58 (42.6)
uCCI	4 (4–6)	4 (4–6)	4 (4–6)	0.600	4 (4–5)
Laboratory parameters					
Hb (mg/dL)	10.8 (8.9–12.9)	11.2 (9.3–13.2)	10.3 (8.5–12.0)	0.068	9.3 (7.7–11.0)^**a**^
WBC (10^3^/µL)	5.8 (3.8–9.5)	5.4 (3.6–9.0)	6.1 (4.4–9.6)	0.235	8.2 (5.5–13.0)^**a**^
Platelets (10^3^/µL)	94 (62–141)	83 (59–134)	104 (64–150)	0.133	89 (63–136)
Creatinine (mg/dL)	(0.7–1.3)	0.9 (0.7–1.2)	1.0 (0.8–1.4)	0.099	0.9 (0.7–1.3)
INR	1.2 (1.1–1.4)	1.2 (1.1–1.5)	1.2 (1.2–1.4)	0.769	1.3 (1.2–1.6)^**a**^
Bilirubin (mg/dL)	2.3 (1.2–3.5)	2.3 (1.2–3.1)	2.3 (1.1–3.9)	0.760	2.2 (1.2–3.6)
Albumin (g/dL)	3.0 (2.8–3.6)	3.3 (2.9–3.7)	2.9 (2.7–3.5)	0.082	2.9 (2.5–3.3)^**a**^
AST (U/L)	74 (50–111)	73 (52–118)	74 (50–111)	0.691	74 (50–133)
ALT (U/L)	41 (27–56)	40 (27–63)	42 (27–56)	0.793	42 (28–67)
Sodium (mmol/L)	138 (134–140)	138 (134–131)	138 (136–140)	0.834	139 (136–142)
NSBB treatment	62 (41.9)	35 (47.3)	27 (36.5)	0.183	60 (44.1)
PPI medication					
Pantoprazole	—	—	71 (95.6)	—	134 (98.5)
Esomeprazole	—	—	2 (2.7)	—	2 (1.5)
Omeprazole	—	—	1 (1.4)	—	0
PPI daily dose^**b**^					
20 mg	—	—	3 (4.1)	—	5 (3.7)
40 mg	—	—	32 (43.2)	—	37 (27.2)
80 mg	—	—	39 (52.7)	—	94 (69.1)
PPI indication^c^					
Gastroduodenal ulcer	—	—	4 (5.4)	—	11 (8.1)
Reflux esophagitis	—	—	4 (5.4)	—	3 (2.2)
Hemorrhagic gastritis	—	—	4 (2.7)	—	8 (5.9)
Variceal bleeding	—	—	15 (20.3)	—	102 (75.0)
Unclear	—	—	47 (63.5)	—	12 (8.8)

a Significantly different against no-PPI group.

b Daily PPI dose at inclusion (pantoprazole dose served as reference: daily doses of omeprazole and esomeprazole equaled double the pantoprazole dose).

c Diagnosed on endoscopy at hospital admission.

Abbreviations: ALT, alanine aminotransferase; AST, aspartate aminotransferase; BCLC, Barcelona clinic liver cancer; CLIF-C AD, chronic liver failure-consortium acute decompensation; Hb, hemoglobin; INR, international normalized ratio; MELD, model for end-stage liver disease; NSBB, nonselective beta-blocker; PPI, proton pump inhibitor; SBP, spontaneous bacterial peritonitis; uCCI, updated Charlson Comorbidity Index; WBC, white blood cell.

### Acute-on-chronic liver failure development and mortality during the observation period

**FIGURE 2:**
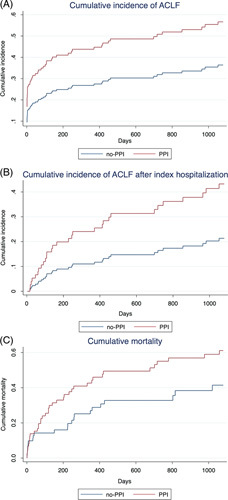
ACLF development and mortality during the observation period. The cumulative incidence of ACLF, considering death as a competing risk, was markedly higher in the PPI group compared with the no-PPI group (A). This result was consistent in a subanalysis including only cases of ACLF or death occurring after the initial hospitalization (B). Cumulative mortality was also significantly higher in the PPI group in comparison to the no-PPI group (C). Abbreviations: ACLF, acute-on-chronic liver failure; PPI, proton pump inhibitor.

In total, 64 patients (43.2%) developed ACLF during the 3-year observation period. A total of 62 patients (41.9%) deceased, thereof 21 patients (14.2%) without prior ACLF development. Indeed, the cumulative incidence of ACLF in the presence of death as competing risk was markedly higher in the PPI group compared with the no-PPI group (0.57 vs 0.36; Figure 2A). Of note, this effect was consistent in a subanalysis including only patients with ACLF or death occurring after the index hospitalization (0.43 vs 0.21; Figure 2B). Cumulative mortality during the observation period was also markedly higher in the PPI group compared with the no-PPI group (0.62 vs 0.42; Figure 2C). ACLF development was associated with comparably poor prognosis in both patient groups, as 3-month mortality after ACLF development was 51.2% in the PPI group and 47.8% in the no-PPI group. Interestingly, PPI treatment was also associated with a higher cumulative incidence of decompensation of cirrhosis without ACLF development (Supplemental Figure S1, http://links.lww.com/HC9/A322). The association of PPI treatment with ACLF could be confirmed in the more permissively 1:2 matched patient collective (Supplemental Figure S2, http://links.lww.com/HC9/A323).

Uni and multivariable competing risk regression models revealed that PPI treatment indeed was an independent predictor of ACLF development (subdistribution HR: 1.892, 95% CI: 1.092–3.281, *p* = 0.023). Other independent predictors of ACLF were variceal bleeding or SBP at study inclusion, white blood cell count, and impaired laboratory parameters of liver function (Table 2). Further, the relevance of daily PPI dose was explored in the regression analyses. In fact, multivariable regression showed that a higher daily PPI dose was independently linked to ACLF development (subdistribution HR: 1.010, 95% CI: 1.002–1.018, *p* = 0.020; (Supplemental Table S2, http://links.lww.com/HC9/A324).

**TABLE 2:** Competing risk regression analyses of predictors of ACLF in the presence of death as a competing risk

	Univariable regression			Multivariable regression		
Parameters	SHR	95% CI	*p*	SHR	95% CI	*p*
Age (y)	1.010	0.999–1.031	0.323	1.019	0.998–1.041	0.069
Sex (M)	1.143	0.669–1.954	0.625	—	—	—
Alcohol-associated liver disease	1.723	1.066–2.787	0.026	1.314	0.780–2.213	0.305
Viral liver disease	0.717	0.376–1.366	0.311	—	—	—
uCCI	1.060	0.923–1.217	0.408	—	—	—
PPI treatment	1.848	1.121–3.044	0.016	1.892	1.092–3.281	0.023
HCC	1.257	0.686–2.301	0.459	—	—	—
Ascites	1.683	0.931–3.043	0.085	0.953	0.519–1.752	0.877
Variceal bleeding	3.076	1.882–5.027	<0.001	2.174	1.241–3.810	0.007
HE	1.418	0.818–2.456	0.213	—	—	—
SBP	2.872	1.530–5.388	0.001	1.904	1.084–3.344	0.025
Bilirubin (mg/dL)	1.066	1.050–1.082	<0.001	1.047	1.017–1.079	0.002
Albumin (g/dL)	0.482	0.347–0.669	<0.001	0.640	0.434–0.944	0.024
INR	4.335	2.550–7.370	<0.001	2.440	1.552–3.836	<0.001
Creatinine (mg/dL)	1.508	1.266–1.796	<0.001	1.175	0.999–1.381	0.051
Sodium (mmol/L)	0.987	0.980–0.994	<0.001	0.987	0.981–0.994	<0.001
WBC (10^3^/µL)	1.061	1.037–1.085	<0.001	1.030	1.008–1.053	0.007
MELD^a^	1.115	1.089–1.141	<0.001	1.024	1.001–1.048	<0.001
CLIF-C AD^a^	1.045	1.025–1.065	<0.001	1.042	1.027–1.057	<0.001

a To avoid bias by variable interference, MELD and CLIF-C AD score and their respective components were entered into multivariable regression separately.

Abbreviations: ACLF, acute-on-chronic liver failure; CLIF-C AD, chronic liver failure-consortium acute decompensation; INR, international normalized ratio; MELD, model for end-stage liver disease; PPI, proton pump inhibitor; SBP, spontaneous bacterial peritonitis; SHR, subdistribution HR; uCCI, updated Charlson Comorbidity Index; WBC, white blood cell.

### Characteristics of acute-on-chronic liver failure in proton pump inhibitor group and no-proton pump inhibitor group

Table 3 presents the features of ACLF stratified according to PPI treatment. In summary, the characteristics of ACLF were not significantly different in the PPI group versus no-PPI group. A majority of patients in both groups developed ACLF grade 1 (n = 26, 40.6%), followed by grade 2 (n = 21, 32.8%) and grade 3 (n = 17, 26.6%). There was no pronounced difference in the pattern of organ dysfunction between the patient groups. ACLF was precipitated by alcohol-associated hepatitis and hemorrhagic shock more often in the no-PPI group, while bacterial infections triggered ACLF more frequently in the PPI group. However, these differences did not reach statistical significance.

**TABLE 3:** Characteristics of ACLF stratified according to PPI treatment

	Patients with ACLF (n = 64)	No PPI treatment (n = 23)	PPI treatment (n = 41)	*p*
Organ failure				
Liver	15 (23.4)	8 (34.8)	7 (17.1)	0.109
Kidney	54 (85.4)	19 (82.6)	35 (85.4)	0.771
Cerebral	14 (21.9)	3 (13.0)	11 (26.8)	0.201
Coagulation	13 (20.3)	3 (13.0)	10 (24.4)	0.279
Respiratory	6 (9.4)	1 (4.3)	5 (12.2)	0.301
Circulatory	22 (34.4)	7 (30.4)	15 (36.6)	0.619
Grade ACLF				0.807
Grade 1	26 (40.6)	10 (43.5)	16 (39.0)	—
Grade 2	21 (32.8)	8 (34.8)	13 (31.7)	—
Grade 3	17 (26.6)	5 (21.7)	12 (29.3)	—
Precipitant factors				
Alcohol-associated hepatitis	23 (35.9)	11 (47.8)	12 (29.3)	0.138
Hemorrhagic shock	12 (18.8)	7 (30.4)	5 (12.2)	0.073
Toxic encephalopathy	4 (6.3)	1 (4.3)	3 (7.3)	0.638
Bacterial infection	11 (17.2)	2 (8.7)	9 (22.0)	0.177
No precipitant	25 (39.1)	8 (34.8)	17 (41.5)	0.599

Abbreviations: ACLF, acute-on-chronic liver failure; PPI, proton pump inhibitor.

### Effect of proton pump inhibitor treatment in patients with high model for end-stage liver disease score and low model for end-stage liver disease score

**FIGURE 3:**
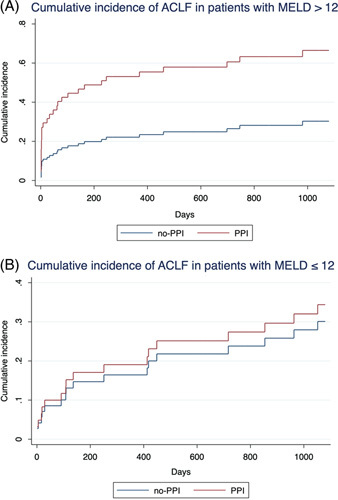
ACLF development in patients with high and low MELD score. The higher cumulative incidence of ACLF in the PPI group compared with the no-PPI group was distinct in patients with a MELD score >12 (A). In contrast, the cumulative incidence of ACLF was not markedly higher in the PPI group versus the no-PPI group in patients with a MELD score ≤12 (B). Abbreviations: ACLF, acute-on-chronic liver failure; MELD, model for end-stage liver disease; PPI, proton pump inhibitor.

Next, the effect of PPI treatment on ACLF development in patients with more impaired liver function and patients with relatively preserved liver function was analyzed. For this purpose, the study collective was stratified into patients with a MELD score > 12, [n = 81, 54.7%, (n = 43 PPI, n = 38 no-PPI)] and patients with a MELD score ≤12 [n = 67, 45.3% (n = 31 PPI, n = 36 no-PPI)]. In fact, the increased cumulative incidence of ACLF in the PPI group compared with the no-PPI group was distinct in patients with a MELD score > 12 (0.66 vs 0.30; Figure 3A). In contrast, the cumulative incidence of ACLF was similar in PPI group and no-PPI group in patients with a MELD score ≤ 12 (0.34 vs 0.30; Figure 3B).

### Bleeding events during the observation period

Besides the identification of ACLF, special focus was put on the occurrence of bleeding complications during the observation period. A total of 33 patients (22.3%) suffered from upper gastrointestinal bleeding (variceal bleeding: n = 23, gastroduodenal ulcer bleeding: n = 5, and banding ulcer bleeding: n = 8). Calculating the cumulative incidence of upper gastrointestinal bleeding, considering death as a competing risk, showed a lower cumulative incidence of bleeding complications in the PPI group in comparison to the no-PPI group (0.24 vs 0.34; Supplemental Figure S3, http://links.lww.com/HC9/A325).

## DISCUSSION

The present study is the first to investigate an impact of PPI treatment on the development of ACLF. Indeed, PPI treatment was associated with markedly increased rates of ACLF in our study collective. This result is of high relevance, as ACLF is a fatal complication with a reported 3-month mortality of up to over 50.0%.^1,14^ In line with these reports, mortality was also strongly linked to the development of ACLF in the present study. ACLF-related deaths contributed substantially to a higher mortality in patients with PPI treatment. This finding is in conformity with a number of previous studies that have reported an association of PPI treatment with increased mortality in patients with cirrhosis.^2,15–17^ The present study is the first to suggest that an increased rate of ACLF may be a central reason for negative prognostic effects of PPI treatment. The increased rate of ACLF in PPI-treated patients was consistent in a subanalysis excluding patients with early ACLF development during the index hospitalization. In this context, it is interesting to note that previous studies have demonstrated microbial effects of PPI initiation and withdrawal already after short-term PPI intake.^7,18^ Assuming that these alterations contribute to adverse effects of PPI treatment in patients with cirrhosis, it cannot be excluded that PPI therapy affects the risk of ACLF already after some days of PPI intake. It is also interesting to note that a higher daily PPI dose was an independent predictor of ACLF in multivariable regression analyses, which suggests that PPI treatment could promote the development of ACLF in a dose-dependent manner. This finding is relevant, since it indicates that not only PPI therapy per se but also dosage may be a decisive factor in the context of ACLF development and thus should be considered when prescribing PPIs. While PPI treatment was associated with an increased occurrence of ACLF in the study collective, it was not linked to the pattern of organ dysfunction, degree of ACLF, or mortality rate following ACLF development. The latter result is in conformity with a recent study that found no significant prognostic effect of PPI treatment in patients admitted with ACLF.^19^ Hemorrhagic shock and alcoholic hepatitis precipitated ACLF more often in the no-PPI group, while bacterial infections precipitated ACLF more frequently in the PPI group, which fits in with previous reports of increased rates of bacterial infections in PPI-treated patients.^3,5^ However, in summary, PPI treatment was not exclusively linked to any precipitating factor of ACLF in particular. This suggests that PPI treatment could be a predisposing factor for the development of ACLF per se. Interestingly, PPI treatment relevantly increased the risk of ACLF only in the subgroup of patients with a MELD score > 12. This finding indicates that PPI treatment is associated with ACLF development especially in patients with advanced cirrhosis. A possible explanation for this phenomenon may be seen in the hypothesis that PPI treatment predisposes for ACLF development through aggravated bacterial translocation and inflammation, as the prevalence of bacterial translocation increases with stage of cirrhosis.^20–22^ However, further fundamental research is needed to identify the pathomechanisms underlying the connection of PPI treatment with ACLF development.

The vast majority of the patients in the PPI group were prescribed PPI treatment in the absence of a clear indication. This finding is in line with previous studies which have reported that prescription of PPIs as (long-term) co-medication is common in patients with cirrhosis.^6,15,23^ To account for possible beneficial effects of PPI treatment, we analyzed the occurrence of upper gastrointestinal bleeding events. Indeed, the cumulative incidence of upper gastrointestinal bleeding was moderately lower in patients receiving PPI treatment. Further, ACLF was precipitated by hemorrhagic shock more often in patients without PPI treatment, albeit the difference compared with PPI-treated patients was not statistically significant. Importantly, these results should be weighed against the increased rates of ACLF and impaired survival in PPI-treated patients in the study collective. Further, it should be noted that international guidelines and consensus recommendations do not support a general prophylactic administration of PPIs to prevent bleeding complications in patients with cirrhosis, as efficacy and safety of PPI medication in this context could not be established in previous studies.^24–26^

The most important limitation of the present study is its retrospective design. This implies that PPI treatment was not assigned in a randomized manner but by physician discretion, which may impair comparability of patient groups. We performed propensity score matching to control for known confounders such as liver function and clinical characteristics of cirrhosis. In addition, we applied uni- and multivariable Cox regression models to consider other predictors of ACLF than PPI treatment. However, of course, it cannot be fully excluded that relevant confounders were not adjusted for. Another aspect that needs to be addressed is assessment of PPI exposure. To account for changes in PPI exposure during the observation period, initiation or withdrawal and dose changes of PPI therapy were considered in the analyses. Due to the study design, PPI medication was not assessed during scheduled follow-up appointments but during intermittent re-hospitalizations and outpatient treatments in clinical routine. Hence, inexact assessment of longitudinal PPI exposure in some cases cannot be fully excluded. Finally, the limited size of the patient collective investigated should be mentioned: After 642 patients were screened initially, 148 patients were included in the primary study collective and 222 patients in the internal validation collective. Overall, these patient numbers were sufficiently high to allow valid analyses. However, in case of some subgroup analyses, for example of particular precipitants of ACLF, the number of patients was rather small. Thus, the results of the present study should be validated in larger, multi-center studies.

In conclusion, the present study is the first to indicate that PPI treatment could be a risk factor for ACLF in patients with cirrhosis. This effect seems to be most pronounced in patients with a higher MELD score. Prospective follow-up studies are needed to clarify the role of PPI treatment in ACLF development. In any case, prescription of PPI treatment should be considered carefully in patients with advanced cirrhosis.

## Supplementary Material
