# Supplementary material for: Proton pump inhibitor treatment is associated with acute-on-chronic liver failure in patients with advanced cirrhosis
Source: Hepatol Commun. 2023 Jun 22;7(7):e00178. doi: 10.1097/HC9.0000000000000178 (PMC10289603; doi:10.1097/HC9.0000000000000178)
Supplement: Supplementary file 1 [file hc9-7-e00178-s001.docx]

**Supplementary table 1: Baseline characteristics of the more permissively 1:2 propensity score matched validation cohort**

|  | **Total of patients**  **(n = 222)** | **No-PPI group**  **(n = 74)** | **PPI group**  **(n = 148)** | **p-value** |
| --- | --- | --- | --- | --- |
| **Gender**  Female  Male | 62 (27.9)  160 (72.1) | 20 (27.0)  54 (73.0) | 42 (28.4)  106 (71.6) | 0.832 |
| **Age** [years] | 60 (51 – 69) | 60 (51 – 69) | 61 (52 – 70) |  |
| **Etiology**  Alcohol-related  HBV  HCV  NASH  Autoimmune  Other/cryptogenic | 106 (47.7)  10 (4.5)  29 (13.1)  11 (5.0)  20 (9.0)  46 (20.7) | 32 (43.2)  5 (6.8)  10 (13.5)  2 (2.7)  8 (10.8)  17 (23.0) | 74 (50.0)  5 (3.4)  19 (12.8)  9 (6.1)  12 (8.1)  29 (19.6) | 0.732 |
| **HCC**  **BCLC stage**  A  B  C | 35 (15.8)  7 (20.0)  18 (51.4)  10 (28.6) | 15 (20.3)  4 (26.7)  4 (26.7)  7 (46.7) | 20 (13.5)  3 (15.0)  14 (70.0)  3 (15.0) | 0.193  0.035 |
| **Variceal bleeding** | 100 (45.0) | 20 (27.0) | 80 (54.1) | < 0.001 |
| **Ascites** | 145 (65.3) | 51 (68.9) | 94 (63.5) | 0.425 |
| **HE** | 52 (23.4) | 17 (23.0) | 35 (23.6) | 0.911 |
| **SBP** | 17 (7.7) | 6 (8.1) | 11 (7.4) | 0.858 |
| **MELD** | 13 (10 – 19) | 13 (10 – 17) | 14 (10 – 20) | 0.064 |
| **CLIF-C AD** | 50 (43 – 60) | 47 (42 – 57) | 51 (44 – 60) | 0.035 |
| **Child-Pugh-Turcotte stage**  A  B  C | 48 (21.6)  100 (45.0)  74 (33.3) | 20 (27.0)  36 (48.6)  18 (24.3) | 28 (18.9)  64 (43.2)  56 (37.8) | 0.104 |
| **uCCI** | 4 (4 – 6) | 4 (4 – 6) | 4 (4 – 6) | 0.737 |
| **Laboratory parameters**  Hb [mg/dl]  WBC [10^3^/µl]  Platelets [10^3^/µl]  Creatinine [mg/dl]  INR  Bilirubin [mg/dl]  Albumin [g/dl]  AST [U/l]  ALT [U/l]  Sodium [mmol/l] | 10.4 (8.5 – 12.7)  6.3 (4.3 – 10.9)  93 (64 – 139)  1.0 (0.7 – 1.4)  1.3 (1.2 – 1.5)  2.3 (1.2 – 3.8)  2.9 (2.8 – 3.5)  74 (50 – 125)  42 (29 – 66)  138 (135 – 141) | 11.2 (9.3 – 13.2)  5.4 (3.6 – 9.0)  83 (59 – 134)  0.9 (0.7 – 1.2)  1.2 (1.1 – 1.5)  2.3 (1.2 – 3.1)  3.3 (2.9 – 3.7)  73 (52 – 118)  40 (27 – 63)  138 (134 – 131) | 10.0 (8.3 – 12.0)  7.0 (4.7 – 12.0)  95 (68 – 142)  1.0 (0.7 – 1.5)  1.3 (1.2 – 1.6)  2.3 (1.2 – 4.2)  2.9 (2.7 – 3.5)  74 (50 – 127)  43 (30 – 72)  138 (136 – 140) | 0.007  0.005  0.197  0.133  0.072  0.641  0.018  0.368  0.249  0.690 |
| **NSBB treatment** | 98 (44.1) | 35 (47.3) | 63 (42.6) | 0.503 |
| **PPI medication**  pantoprazole  esomeprazole  omeprazole |  |  | 144 (97.3)  3 (2.0)  1 (0.7) |  |
| **PPI daily dose^a^**  20 mg  40 mg  80 mg |  |  | 7 (4.7)  56 (37.8)  84 (56.8) |  |
| **PPI indication^b^**  Gastroduodenal ulcer  Reflux esophagitis  Hemorrhagic gastritis  Variceal bleeding  Unclear |  |  | 10 (6.8)  5 (3.4)  7 (4.7)  66 (44.6)  88 (59.5) |  |

^a^ Daily PPI dose at inclusion. Pantoprazole dose served as reference: Daily doses of omeprazole and esomeprazole equaled double the pantoprazole dose.

^b^ Diagnosed on endoscopy at hospital admission.

Abbreviations: ALT – alanine aminotransferase, AST – aspartate aminotransferase, BCLC – Barcelona Clinic Liver Cancer, CLIF-C AD – Chronic Liver Failure-Consortium Acute Decompensation (score), Hb – hemoglobin, HBV/HCV – chronic hepatitis B/C virus infection, HCC – hepatocellular carcinoma, HE – hepatic encephalopathy, INR – international normalized ratio, MELD – Model for End-Stage Liver Disease, NASH – non-alcohol-related steatohepatitis, NSBB – non-selective beta blocker, PPI – proton pump inhibitor, SBP – spontaneous bacterial peritonitis, uCCI – updated Charlson Comorbidity Index, WBC – white blood cells
