# Supplementary figures and images for: Proton pump inhibitor treatment is associated with acute-on-chronic liver failure in patients with advanced cirrhosis
Source: Hepatol Commun. 2023 Jun 22;7(7):e00178. doi: 10.1097/HC9.0000000000000178 (PMC10289603; doi:10.1097/HC9.0000000000000178)

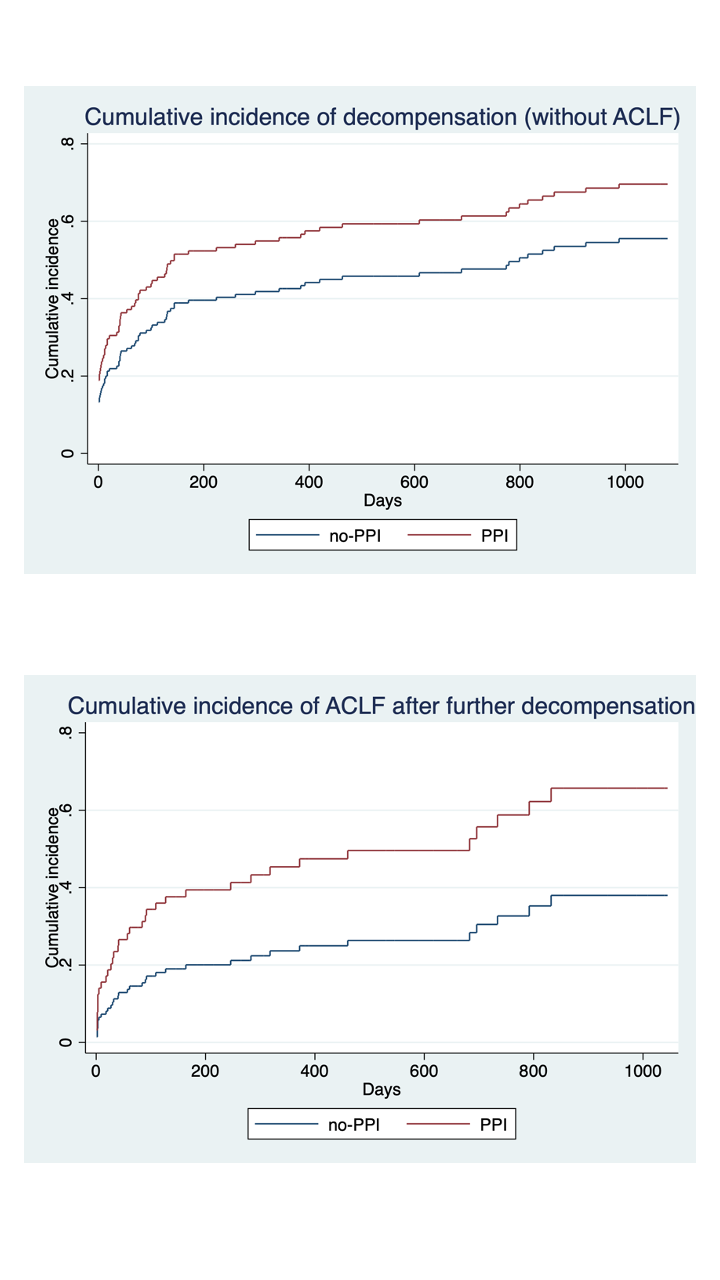

Supplement: Supplementary file 2 [file hc9-7-e00178-s002.tiff]

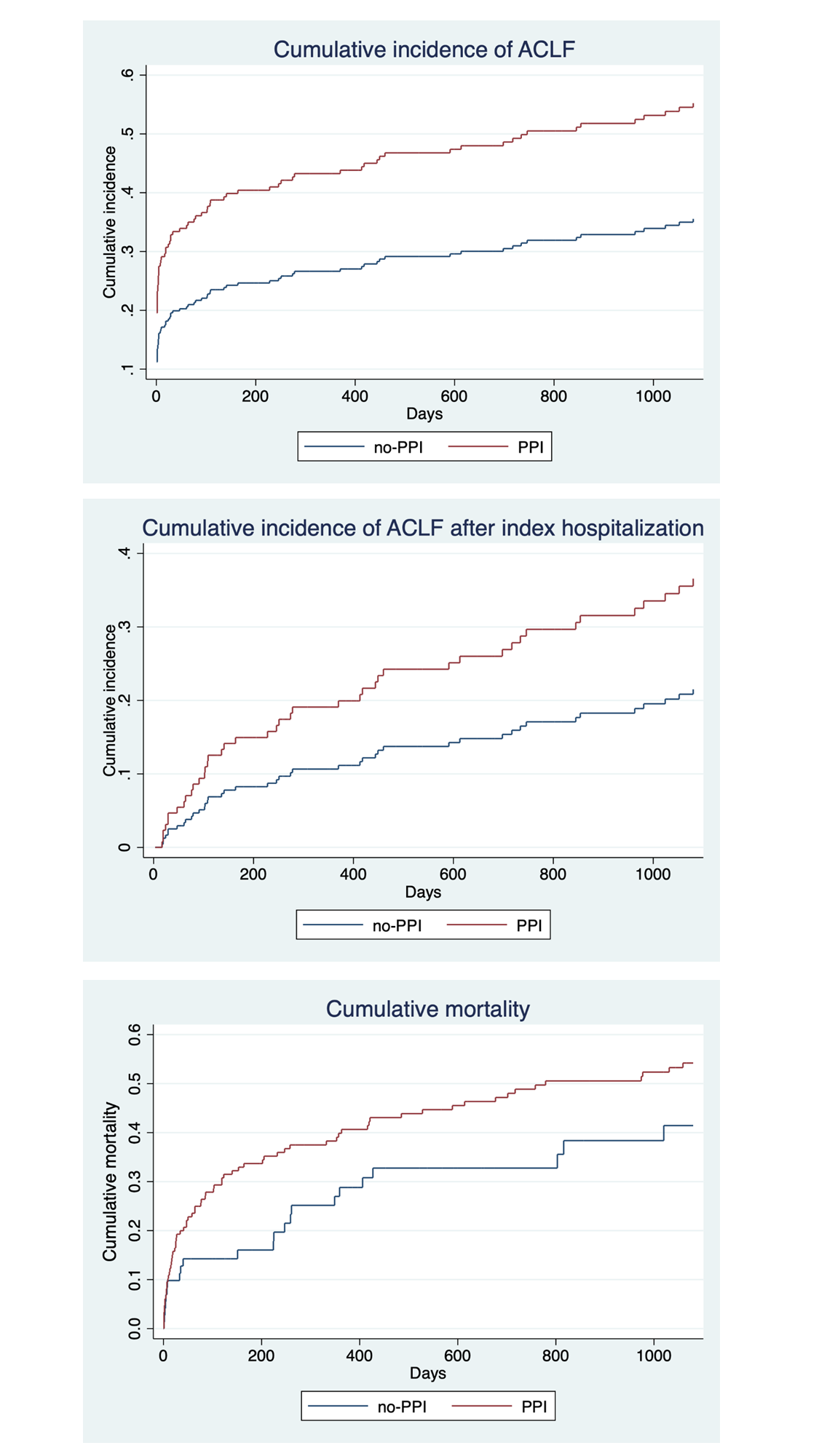

Supplement: Supplementary file 3 [file hc9-7-e00178-s003.tif]

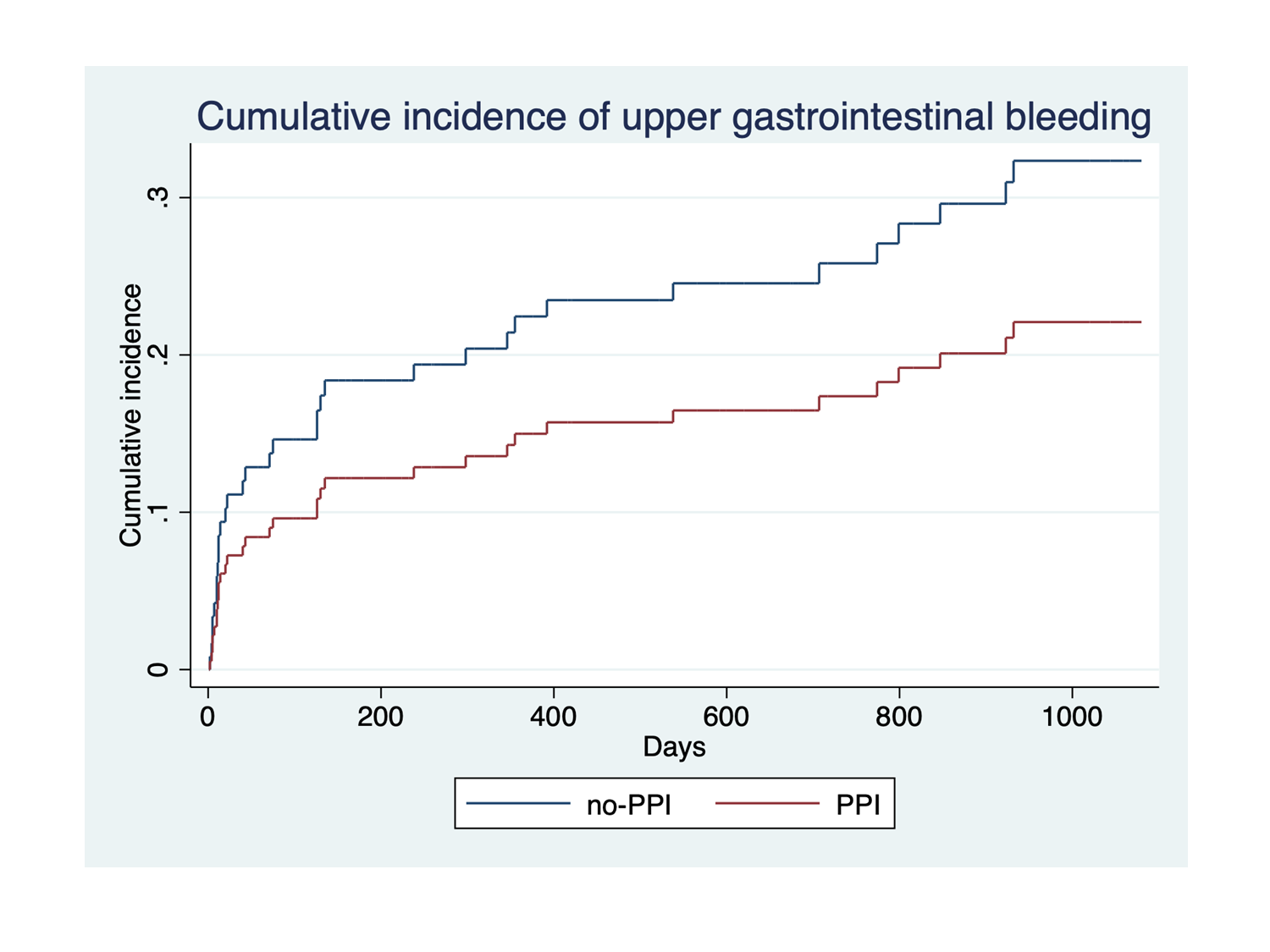

Supplement: Supplementary file 5 [file hc9-7-e00178-s005.tif]
