# Supplementary material for: Proton pump inhibitor treatment is associated with acute-on-chronic liver failure in patients with advanced cirrhosis
Source: Hepatol Commun. 2023 Jun 22;7(7):e00178. doi: 10.1097/HC9.0000000000000178 (PMC10289603; doi:10.1097/HC9.0000000000000178)
Supplement: Supplementary file 4 [file hc9-7-e00178-s004.docx]

**Supplementary table 2: Competing risk regression analyses of predictors of ACLF in the presence of death as competing risk, including daily PPI dose**

|  | **Univariable regression** | | | **Multivariable regression** | | |
| --- | --- | --- | --- | --- | --- | --- |
| **Parameters** | **SHR** | **95 % CI** | **p-value** | **SHR** | **95 % CI** | **p-value** |
| Age [years] | 1.010 | 0.999 – 1.031 | 0.323 | 1.021 | 1.001 – 1.042 | 0.043 |
| Male gender | 1.143 | 0.669 – 1.954 | 0.625 |  |  |  |
| Alcohol-related liver disease | 1.723 | 1.066 – 2.787 | 0.026 | 1.305 | 0.776 – 2.195 | 0.315 |
| Viral liver disease | 0.717 | 0.376 – 1.366 | 0.311 |  |  |  |
| uCCI | 1.060 | 0.923 – 1.217 | 0.408 |  |  |  |
| PPI daily dose [mg] | 1.010 | 1.003 – 1.017 | 0.003 | 1.010 | 1.002 – 1.018 | 0.020 |
| HCC | 1.257 | 0.686 – 2.301 | 0.459 |  |  |  |
| Ascites | 1.683 | 0.931 – 3.043 | 0.085 | 0.991 | 0.538 – 1.828 | 0.978 |
| Variceal bleeding | 3.076 | 1.882 – 5.027 | < 0.001 | 2.106 | 1.210 – 3.666 | 0.008 |
| HE | 1.418 | 0.818 – 2.456 | 0.213 |  |  |  |
| SBP | 2.872 | 1.530 – 5.388 | 0.001 | 2.051 | 1.089 – 3.861 | 0.026 |
| Bilirubin [mg/dl] | 1.066 | 1.050 – 1.082 | < 0.001 | 1.053 | 1.023 – 1.084 | < 0.001 |
| Albumin [g/dl] | 0.482 | 0.347 – 0.669 | < 0.001 | 0.657 | 0.444 – 0.972 | 0.036 |
| INR | 4.335 | 2.550 – 7.370 | < 0.001 | 2.382 | 1.486 – 3.818 | < 0.001 |
| Creatinine [mg/dl] | 1.508 | 1.266 – 1.796 | < 0.001 | 1.145 | 0.973 – 1.348 | 0.103 |
| Sodium [mmol/l] | 0.987 | 0.980 – 0.994 | < 0.001 | 0.987 | 0.981 – 0.994 | < 0.001 |
| WBC [10^3^/µl] | 1.061 | 1.037 – 1.085 | < 0.001 | 1.027 | 1.004 – 1.050 | 0.019 |

Abbreviations: ACLF – acute-on-chronic liver failure, CI – confidence interval, HCC – hepatocellular carcinoma, HE – hepatic encephalopathy, INR – international normalized ratio, PPI – proton pump inhibitor, SBP – spontaneous bacterial peritonitis, SHR – subdistribution hazard ratio, uCCI – updated Charlson Comorbidity Index, WBC – white blood cells
